# Precision Medicine in Acute Coronary Syndromes: Integrating Plaque Phenotyping, Personalized Revascularization, Antithrombotic Strategies, and Residual Risk Reduction

**DOI:** 10.3390/jcm15218248

**Published:** 2026-10-23

**Authors:** Ahmad Omari, Yazeed Omari, Ahmad Kharoufeh, Mohamedanas Mohamedfaruk Patni, Thilakavathy Pandurangan, Abdalla Ahmed Eldaw Elamin, Ramya Kundayi Ravi, Omar Emad Khater, Mohammadnour Malkawi, Omar Aldwiri

**Affiliations:** 1 RAK College of Medical Sciences, Ras Al Khaimah Medical and Health Sciences University, Ras Al Khaimah 11172, United Arab Emirates; ahmad.21901070@rakmhsu.ac.ae (A.O.); ahmad.21901103@rakmhsu.ac.ae (A.K.); omar.23901066@rakmhsu.ac.ae (O.E.K.); mohammadnour.23901050@rakmhsu.ac.ae (M.M.); omar.23901117@rakmhsu.ac.ae (O.A.); 2 Department of Community Medicine, RAK College of Medical Sciences, Ras Al Khaimah Medical and Health Sciences University, Ras Al Khaimah 11172, United Arab Emirates; mohamedanas@rakmhsu.ac.ae; 3 Department of Microbiology, RAK College of Medical Sciences, Ras Al Khaimah Medical and Health Sciences University, Ras Al Khaimah 11172, United Arab Emirates; 4 Department of Anatomy, RAK College of Medical Sciences, Ras Al Khaimah Medical and Health Sciences University, Ras Al Khaimah 11172, United Arab Emirates; abdalla@rakmhsu.ac.ae; 5 RAK College of Nursing, Ras Al Khaimah Medical and Health Sciences University, Ras Al Khaimah 11172, United Arab Emirates; ramya@rakmhsu.ac.ae

**Keywords:** acute coronary syndrome, precision medicine, intracoronary imaging, revascularization, antithrombotic therapy, residual cardiovascular risk, secondary prevention

## Abstract

Acute coronary syndromes (ACS) are biologically and clinically heterogeneous, with substantial variation in mechanisms of coronary instability, coronary anatomy, ischemic and bleeding risk, and residual cardiovascular risk. Although advances in imaging, risk stratification, and targeted therapies have expanded opportunities for individualized care, their clinical actionability remains uneven. This narrative review proposes a longitudinal precision-medicine framework integrating four interconnected domains—mechanism, anatomy, ischemic–bleeding risk, and residual biological risk—to examine how patient characterization can inform treatment selection and reassessment. Contemporary evidence on mechanistic phenotyping, coronary imaging, revascularization, antithrombotic therapy, and residual cardiovascular risk is critically synthesized, with particular emphasis on distinguishing strategies supported by randomized clinical-outcome evidence from those supported primarily by mechanistic, imaging, biomarker, or other surrogate endpoints. Mechanistic and imaging-based characterization can refine ACS phenotyping, while coronary anatomy and clinical characteristics inform revascularization decisions. Dynamic assessment of ischemic and bleeding risk supports individualized antithrombotic strategies, and lipid-related, inflammatory, thrombotic, and cardiometabolic phenotypes inform residual-risk assessment and secondary prevention. However, the evidence supporting phenotype-guided treatment remains heterogeneous. Several anatomy- and risk-informed strategies have demonstrated clinical benefit, whereas morphology-guided intervention, biomarker-directed treatment, molecular phenotyping, and artificial-intelligence-assisted decision support require further validation before routine implementation. The proposed framework distinguishes clinically actionable information from findings that remain primarily prognostic or hypothesis-generating. Diagnostic precision currently exceeds therapeutic precision in several areas of ACS care. A longitudinal framework integrating mechanism, anatomy, ischemic–bleeding risk, and residual biological risk may help connect patient phenotyping to evidence-based treatment decisions, identify where treatment-effect evidence remains insufficient, and support reassessment as clinical and biological risk evolves. Precision ACS care should therefore prioritize demonstrated clinical benefit rather than diagnostic sophistication alone.

## 1. Introduction

ACS remain a major cause of cardiovascular morbidity and mortality despite advances in reperfusion, invasive management, antithrombotic therapy, and secondary prevention [1,2]. Although ACS encompasses ST-segment elevation myocardial infarction (STEMI), non-ST-segment elevation myocardial infarction (NSTEMI), and unstable angina, these clinical categories do not fully capture the heterogeneity relevant to treatment decisions. The recently published Fifth Universal Definition of Myocardial Infarction (2026) further emphasizes the importance of distinguishing myocardial injury from myocardial infarction (MI) and classifying infarction according to its underlying pathophysiological mechanism [3]. Within this updated framework, primary MI, secondary MI, and procedure-related MI represent distinct diagnostic categories that complement the clinical classification of ACS. Patients with similar presentations may therefore differ substantially in the mechanism of coronary instability, coronary anatomy, ischemic and bleeding risk, and residual cardiovascular risk after stabilization.

Advances in diagnostic technologies have improved characterization of this heterogeneity. High-sensitivity cardiac troponin enhances detection of myocardial injury, although elevated troponin concentrations alone do not establish a diagnosis of MI without supporting evidence of acute myocardial ischemia [1,3,4]. Optical coherence tomography (OCT), intravascular ultrasound (IVUS), and cardiovascular magnetic resonance (CMR) provide additional insights into coronary and myocardial pathology [1,2]. Potential mechanisms include plaque rupture, plaque erosion, calcified nodules, spontaneous coronary artery dissection (SCAD), coronary embolism, vasospasm, and microvascular dysfunction. The updated diagnostic framework reinforces the need to integrate clinical presentation, electrocardiographic findings, cardiac biomarkers, and imaging evidence when distinguishing ischemic infarction from nonischemic myocardial injury and identifying the predominant underlying mechanism [3]. However, identifying a mechanism does not necessarily establish the clinical benefit of a corresponding mechanism-specific intervention.

Treatment decisions extend beyond diagnostic classification. Revascularization depends on clinical stability, coronary anatomy, lesion complexity, and procedural risk, while antithrombotic therapy requires balancing protection against recurrent ischemia with bleeding risk [1,2]. Following stabilization, residual cardiovascular risk may reflect persistent lipid-related, inflammatory, thrombotic, and cardiometabolic abnormalities. These considerations create opportunities for individualized management throughout the ACS continuum, although the clinical value of such strategies depends on the evidence supporting their application.

In this review, precision medicine is defined in a clinically integrated sense rather than being restricted to genomic or molecular stratification. We conceptualize precision ACS care as the use of reproducible mechanistic, anatomical, clinical, and biological characteristics to inform treatment selection or modification when supported by evidence of their relevance to the expected balance of benefit and harm. Importantly, three distinct levels of precision must be differentiated: diagnostic precision, which improves identification of the underlying disease mechanism or phenotype; prognostic precision, which refines the estimation of future cardiovascular risk; and therapeutic precision, which requires evidence that patient characteristics can meaningfully guide treatment selection to improve clinical outcomes. Although diagnostic and prognostic advances may inform clinical decision-making, they do not necessarily establish that a particular phenotype predicts differential benefit from a specific intervention. Distinguishing these levels is therefore essential when evaluating the clinical actionability of precision strategies.

Previous reviews have examined precision medicine in ACS through mechanistic phenotyping, intracoronary imaging, biomarker assessment, and individualized management [5]. Building on this literature, the principal contribution of the present review is its explicit focus on the distinction between improved patient characterization and demonstrated treatment benefit. Rather than primarily cataloging emerging diagnostic technologies or therapeutic possibilities, this review critically evaluates whether mechanistic, anatomical, clinical, and biological information can support actionable treatment decisions, the strength of the corresponding treatment-effect evidence, and the need for longitudinal reassessment. In doing so, it seeks to clarify where precision medicine has already translated into evidence-based clinical practice and where therapeutic precision remains an investigational objective.

To achieve this, we integrate precision ACS care within four interconnected conceptual domains: (1) the mechanism of coronary instability, (2) coronary anatomy and revascularization requirements, (3) the evolving balance between ischemic and bleeding risk, and (4) residual biological risk after stabilization. These domains provide a framework for connecting biological and clinical heterogeneity with diagnostic assessment, revascularization, antithrombotic treatment, and secondary prevention across the ACS continuum.

The proposed framework emphasizes the distinction between strategies supported by randomized clinical-outcome evidence, approaches informed primarily by diagnostic or prognostic findings, and interventions supported predominantly by mechanistic or surrogate-endpoint evidence. It is intended as an organizing synthesis rather than a prospectively validated treatment algorithm. By critically examining clinical actionability and evidentiary limitations across the four domains, this review aims to identify where individualized strategies can improve evidence-based ACS management, where enhanced phenotyping has not yet translated into demonstrated therapeutic benefit, and which questions require further investigation. Figure 1 illustrates the proposed longitudinal framework and its relationship to treatment decisions across the ACS continuum.

## 2. Review Approach and Evidence Selection

This narrative review provides a contemporary, clinically oriented synthesis of precision medicine in acute coronary syndromes (ACSs). The review progresses from the biological and clinical heterogeneity of ACSs to precision diagnosis and multimodal phenotyping, individualized revascularization, personalized antithrombotic therapy, and the management of residual cardiovascular risk. These topics are integrated within a longitudinal precision-medicine framework encompassing four interconnected conceptual domains: the mechanism of coronary instability, coronary anatomy and revascularization requirements, the evolving balance between ischemic and bleeding risk, and residual biological risk after stabilization. Coronary imaging is addressed across the mechanistic, diagnostic, and interventional discussions because of its complementary roles in lesion characterization, diagnostic clarification, and procedural optimization. This organization connects biological mechanisms and diagnostic findings with evidence-based therapeutic decisions and longitudinal risk reassessment.

Relevant literature was identified through topic-focused searches of PubMed, supplemented by examination of reference lists from relevant clinical practice guidelines and major reviews. The principal search concepts included acute coronary syndromes, myocardial infarction, precision medicine, plaque rupture, plaque erosion, coronary thrombosis, intracoronary imaging, optical coherence tomography, intravascular ultrasound, cardiovascular magnetic resonance, revascularization, cardiogenic shock, mechanical circulatory support, dual antiplatelet therapy, ischemic risk, bleeding risk, lipid-lowering therapy, inflammation, cardiometabolic risk, and secondary prevention. Search concepts were considered according to the specific topics addressed in the review, with emphasis on contemporary literature and inclusion of earlier landmark studies when necessary to establish important pathophysiological or therapeutic concepts. Because the searches were undertaken for a narrative synthesis rather than according to a prespecified systematic-review protocol, a reproducible search date range and formal publication-date restrictions were not documented.

Evidence selection prioritized contemporary international clinical practice guidelines, landmark and recent randomized controlled trials, systematic reviews and meta-analyses, and large prospective studies relevant to the principal topics covered in the review. Earlier influential studies were included when necessary to establish important pathophysiological or therapeutic concepts. Mechanistic and imaging studies were considered when they contributed clinically relevant information regarding ACS phenotyping, diagnostic refinement, or potential treatment selection.

The synthesis emphasized the clinical actionability of patient characteristics and the strength of evidence supporting corresponding treatment decisions. Particular attention was given to distinguishing interventions supported by clinical-outcome data from approaches supported primarily by mechanistic, imaging, biomarker, or other surrogate-endpoint evidence. Where relevant, differences in study populations, treatment strategies, endpoints, and follow-up were considered when interpreting findings across studies. Diagnostic and prognostic information was distinguished from evidence demonstrating that a particular phenotype predicts differential benefit from an intervention, thereby avoiding the assumption that improved patient characterization necessarily translates into improved clinical outcomes.

To make the strength of supporting evidence explicit, strategies were characterized using four complementary descriptors: guideline-supported, randomized clinical-outcome evidence, surrogate-endpoint evidence, and investigational. Guideline-supported strategies are those endorsed by contemporary practice guidelines for specified clinical circumstances. Randomized clinical-outcome evidence refers to trials evaluating clinically meaningful endpoints, whereas surrogate-endpoint evidence primarily concerns imaging, physiological, or biomarker outcomes without definitive clinical-outcome validation. Investigational approaches require further prospective evaluation before routine use. These descriptors are not mutually exclusive and refer to the specific clinical application rather than the overall maturity of a technology or treatment class.

As a narrative rather than systematic review, this article was not designed to achieve exhaustive literature identification, formal study-level risk-of-bias assessment, or quantitative evidence synthesis. The selected literature was critically organized to identify where patient characteristics can inform evidence-based management, where evidence remains insufficient to establish phenotype-guided treatment benefit, and which questions require further investigation. The following sections examine the biological mechanisms underlying ACS, the diagnostic and therapeutic implications of patient heterogeneity, and the strength of evidence supporting individualized management strategies across the disease continuum.

## 3. Biological and Clinical Heterogeneity of Acute Coronary Syndromes

### 3.1. Plaque Rupture

Plaque rupture is a major pathological substrate of ACS [6,7], although its reported prevalence varies across populations, clinical presentations, and diagnostic methods. Disruption of the fibrous cap exposes the underlying thrombogenic plaque contents to circulating blood, promoting platelet activation, coagulation, and thrombus formation. Rupture-prone plaques commonly exhibit a large lipid-rich necrotic core, a thin fibrous cap, and inflammatory-cell infiltration [6].

Optical coherence tomography (OCT) enables in vivo identification of plaque rupture by demonstrating fibrous-cap discontinuity, often with an underlying cavity and associated thrombus [7]. These findings can clarify the culprit-lesion mechanism and inform interpretation of coronary anatomy, thrombus burden, and procedural requirements.

### 3.2. Plaque Erosion

Plaque erosion is characterized by coronary thrombosis over an intact fibrous cap, without the structural disruption observed in plaque rupture, and may account for approximately one-third of ACS presentations in some studied populations [7,8]. Compared with ruptured plaques, eroded lesions generally exhibit a preserved fibrous cap, a smaller or absent necrotic core, and less extensive structural disruption. Proposed mechanisms include disturbed endothelial shear stress, endothelial dysfunction, inflammatory activation, and neutrophil extracellular trap formation [8].

OCT may suggest plaque erosion when thrombus or an irregular luminal surface is present without visible fibrous-cap rupture. However, OCT-defined erosion remains an indirect diagnosis because endothelial-cell loss cannot be visualized directly [7,8]. Proof-of-concept studies suggest that selected patients with OCT-defined erosion, adequate residual lumen, and preserved coronary flow may be managed with intensive antithrombotic therapy without immediate stent implantation [9]. The clinical implications and evidentiary limitations of this approach are considered in Section 3.7.

### 3.3. Calcified Nodules

Calcified nodules are an uncommon substrate of coronary thrombosis and are generally associated with advanced calcific atherosclerosis [6,7]. Eruptive calcified nodules involve fragmented calcium protruding into the coronary lumen through disrupted overlying tissue and are frequently accompanied by thrombus. Pathological studies have reported calcified nodules in approximately 2–7% of fatal coronary thrombosis cases [6], although these estimates should not be extrapolated directly to unselected ACS populations.

OCT can characterize nodular calcium, including features suggestive of eruptive and noneruptive morphology, as well as the extent and distribution of calcification [7]. Such information may influence procedural planning because severe, irregular calcium can complicate lesion preparation and impair stent expansion or apposition.

### 3.4. Spontaneous Coronary Artery Dissection

Spontaneous coronary artery dissection (SCAD) is a nonatherosclerotic cause of ACS involving intramural hematoma and/or disruption of the coronary arterial wall, resulting in compression of the true lumen [10]. It disproportionately affects younger and middle-aged women and is associated with underlying arteriopathies, including fibromuscular dysplasia [10]. Recognition is clinically important because its management may differ substantially from that of conventional atherothrombotic ACS.

Coronary angiography is the principal diagnostic modality. OCT or intravascular ultrasound (IVUS) may help clarify uncertain cases but should be used selectively because coronary instrumentation can propagate dissection or compromise the true lumen [10]. In clinically stable patients without ongoing ischemia or high-risk anatomical features, conservative management is generally favored because spontaneous healing is common and percutaneous coronary intervention (PCI) can be technically challenging. Revascularization may be necessary in patients with ongoing ischemia, hemodynamic instability, or high-risk anatomy [10]. The optimal antiplatelet regimen for conservatively managed SCAD remains uncertain.

### 3.5. Coronary Vasospasm and Coronary Embolism

Coronary vasospasm and embolism are alternative mechanisms of ACS whose recognition can redirect management away from routine treatment of an assumed atherosclerotic culprit lesion.

Vasospasm involves transient, intense epicardial coronary constriction associated with abnormalities in vascular smooth-muscle reactivity, endothelial function, autonomic regulation, and vasomotor control [11]. In appropriately selected patients, provocative testing can support the diagnosis when the clinical presentation and coronary findings raise suspicion. Treatment is directed primarily toward vasodilator therapy, particularly calcium-channel blockers, with nitrates used according to the clinical context [11].

Coronary embolism may arise from atrial fibrillation, intracardiac thrombus, prosthetic valves, infective endocarditis, paradoxical embolism, or other embolic sources [12]. Angiographic findings such as abrupt vessel occlusion with limited underlying atherosclerosis, distal obstruction, or involvement of multiple coronary territories may raise suspicion. Intracoronary imaging may help clarify the mechanism when diagnostic uncertainty persists. Catheter-based intervention may be required to restore coronary flow in selected cases, but routine stenting may be unnecessary when no underlying atherosclerotic culprit lesion is present. Subsequent management should address the identified embolic source and its underlying cause [12].

### 3.6. Coronary Microvascular Dysfunction

Coronary microvascular dysfunction (CMD) encompasses abnormalities of the coronary microcirculation that may contribute to myocardial ischemia when obstructive epicardial coronary disease does not adequately explain the clinical presentation [13]. Potential mechanisms include impaired vasodilation, increased microvascular resistance, microvascular spasm, inflammation, oxidative stress, and adverse microvascular remodeling. CMD may coexist with obstructive coronary atherosclerosis and should not be considered synonymous with myocardial infarction with non-obstructive coronary arteries (MINOCA) [13].

When clinically suspected, CMD may be assessed using invasive coronary function testing, including coronary flow reserve, indices of microvascular resistance, and acetylcholine provocation, while positron emission tomography and stress cardiovascular magnetic resonance can provide complementary noninvasive assessment [13]. Recognition may help identify a mechanism of ischemia that is not amenable to epicardial stenting. CMD nevertheless encompasses multiple endotypes, and the evidence supporting specific endotype-directed therapies in ACS remains limited.

### 3.7. From Mechanistic Phenotyping to Therapeutic Precision

Mechanistic phenotyping can inform clinical management, but the strength of evidence supporting mechanism-directed interventions varies substantially. Recognition of spontaneous coronary artery dissection (SCAD) may favor conservative management in stable patients [10], vasospasm directs vasodilator therapy [11], and coronary embolism requires evaluation and treatment of the embolic source [12]. In contrast, plaque morphology provides important diagnostic and procedural information without necessarily establishing a distinct treatment pathway.

Recent evidence supports a broader causal endotype–based approach to myocardial infarction (MI) classification. In a prospective multicenter registry study involving 6282 patients, Armillotta et al. classified MI according to four predominant etiological categories: cardiac/coronary (84.9%), cardiac/noncoronary (4.8%), systemic (8.0%), and indeterminate (2.3%) [14]. One-year all-cause mortality differed across these groups, occurring in 9.8%, 15.9%, 25.8%, and 1.4% of patients, respectively. After adjustment, systemic and cardiac/noncoronary endotypes were associated with higher all-cause mortality than the cardiac/coronary group (hazard ratios, 2.50 and 1.46, respectively). These differences were largely attributable to noncardiovascular mortality, whereas differences in major adverse cardiovascular events were less pronounced. The findings suggest that causal endotype classification can identify clinically distinct populations not fully characterized by the conventional type 1/type 2 MI framework [14].

This approach is consistent with the mechanistic emphasis of the Fifth Universal Definition of Myocardial Infarction (2026), which distinguishes primary, secondary, and procedure-related MI [3]. However, the registry study was observational and applied the earlier Fourth Universal Definition; its endotype categories should therefore be viewed as a complementary research framework rather than an independently validated replacement for the updated clinical classification. Importantly, the observed prognostic differences do not establish that endotype-guided treatment improves clinical outcomes.

For example, OCT-defined plaque erosion has motivated no-stent strategies in carefully selected patients [9], but definitive randomized clinical-outcome evidence remains insufficient for routine adoption. Similarly, identifying extensive calcification can guide lesion preparation, whereas treatment selection based specifically on calcified-nodule subtype remains less well established.

The clinical application of mechanistic phenotyping should therefore distinguish established management implications from emerging strategies and integrate coronary anatomy, procedural feasibility, ischemic and bleeding risk, and treatment-effect evidence.

Figure 2 summarizes the relationship between major ACS mechanisms, the diagnostic modalities used to identify them, and the current clinical actionability of mechanism-guided management.

## 4. Precision Diagnosis and Multimodal Phenotyping in Acute Coronary Syndromes

### 4.1. High-Sensitivity Cardiac Troponin and Accelerated Diagnostic Pathways

High-sensitivity cardiac troponin (hs-cTn) assays enable earlier detection of myocardial injury and support accelerated diagnostic pathways in suspected ACS [1,2,4]. The Fifth Universal Definition of Myocardial Infarction (2026) reinforces the distinction between myocardial injury and myocardial infarction (MI), emphasizing that elevated cardiac troponin concentrations indicate myocardial injury but do not independently establish an ischemic mechanism [3]. Myocardial injury is identified by cardiac troponin concentrations exceeding the assay-specific 99th-percentile upper reference limit, with a rise and/or fall indicating acute injury. A diagnosis of acute MI additionally requires evidence of acute myocardial ischemia, assessed through clinical presentation, electrocardiographic findings, imaging, or other relevant diagnostic information [3,4].

The updated definition emphasizes classification according to the predominant underlying mechanism, distinguishing primary MI, secondary MI, and procedure-related MI [3]. Primary MI reflects an acute coronary pathology, including atherothrombosis, spontaneous coronary artery dissection, coronary embolism, or vasospasm. Secondary MI results from myocardial oxygen supply–demand imbalance caused by another acute condition, rather than a primary acute coronary pathology. Procedure-related MI is considered separately. These categories provide a mechanistic framework for interpreting troponin elevation, although clinical assessment remains essential because acute non-ischemic myocardial injury may occur in conditions such as myocarditis, sepsis, acute heart failure, and other systemic illnesses. Accordingly, elevated hs-cTn should prompt consideration of both the presence of ischemia and the underlying cause of myocardial injury rather than automatic classification as ACS [3,4].

ESC 0/1-h and 0/2-h algorithms combine assay-specific hs-cTn concentrations and serial changes to classify patients into rule-out, observation, and rule-in categories [2,15]. A single very low value may permit rule-out in appropriately selected patients presenting sufficiently long after symptom onset. Early presenters and patients in the observation zone require repeat testing and clinical reassessment [2,15]. These algorithms support rapid identification of patients requiring further evaluation but do not independently determine the underlying mechanism of myocardial injury.

Interpretation should account for age, kidney function, structural heart disease, systemic illness, and biological differences between women and men. The Fifth Universal Definition further highlights the importance of assay-specific and sex-specific 99th-percentile thresholds where appropriately validated [3]. Accelerated pathways can facilitate earlier discharge of selected low-risk patients but must not override persistent ischemic symptoms, concerning electrocardiographic findings, hemodynamic instability, or high clinical suspicion [1,2,15]. When the mechanism remains uncertain, clinical assessment and targeted imaging are necessary to distinguish ischemic infarction from non-ischemic myocardial injury and identify potentially actionable causes.

### 4.2. Intracoronary Imaging: From Morphological Diagnosis to PCI Optimization

Coronary angiography defines luminal anatomy but provides limited information about plaque morphology and vessel-wall architecture. Optical coherence tomography (OCT) and intravascular ultrasound (IVUS) offer complementary information for culprit-lesion assessment and percutaneous coronary intervention (PCI) optimization [2,7]. Within the updated mechanistic classification of MI, intracoronary imaging may also help clarify whether an acute coronary lesion accounts for the clinical presentation when angiographic findings are inconclusive [3].

#### 4.2.1. Complementary Roles of OCT and IVUS

OCT provides high spatial resolution for assessing fibrous-cap disruption, thrombus, superficial calcium, dissection, and stent-related abnormalities [7,16]. IVUS offers greater tissue penetration and is useful for evaluating vessel dimensions, plaque burden, deeper calcium, remodeling, and large or complex coronary segments, including left-main disease [16]. Modality selection should therefore reflect the diagnostic or procedural question.

When angiography leaves the culprit mechanism uncertain, OCT may identify plaque rupture, features compatible with erosion, or calcified nodules, while IVUS can clarify plaque burden, calcification, and vessel architecture [7,16]. These findings refine anatomical and mechanistic assessment but do not independently establish the benefit of a morphology-specific treatment strategy.

#### 4.2.2. Imaging-Guided PCI and Clinical Evidence

Intracoronary imaging can guide lesion preparation, stent sizing, landing-zone selection, and identification of underexpansion, malapposition, edge dissection, or residual disease.

Randomized trials support imaging guidance in selected complex PCI settings. RENOVATE-COMPLEX-PCI reported a lower incidence of its primary composite endpoint with intravascular imaging-guided versus angiography-guided PCI (7.7% vs. 12.3%; hazard ratio [HR], 0.64) [17]. OCTOBER similarly reported improved outcomes with OCT-guided PCI for complex bifurcation lesions (10.1% vs. 14.1%; HR, 0.70) [18].

Results are not uniform across trials or endpoints. In ILUMIEN IV, OCT guidance increased minimum stent area and reduced stent thrombosis but did not significantly reduce the primary endpoint of target-vessel failure (7.4% vs. 8.2%; HR, 0.90) [19]. These findings support evaluating procedural improvements alongside, rather than as substitutes for, clinical outcomes.

#### 4.2.3. Clinical Integration

OCT or IVUS selection should consider lesion complexity, diagnostic uncertainty, procedural objectives, renal function, hemodynamic stability, and operator expertise [2,16]. OCT generally requires contrast-mediated blood clearance, whereas IVUS may be preferable when contrast exposure should be minimized or deeper vessel assessment is needed [16].

Imaging-guided PCI has randomized clinical-outcome evidence in selected settings [17,18,19]. In contrast, changing treatment solely according to plaque morphology—such as routinely avoiding stenting in OCT-defined erosion—remains less well validated [7,9]. Thus, intracoronary imaging has complementary roles in mechanistic diagnosis and procedural optimization, but the strength of evidence supporting treatment modification differs between these applications.

### 4.3. Multimodality Assessment in Myocardial Injury with Non-Obstructive Coronary Arteries and Uncertain Culprit Lesions

Patients presenting with acute myocardial injury or suspected infarction despite non-obstructive coronary arteries represent a diagnostically heterogeneous population. Historically, myocardial infarction with non-obstructive coronary arteries (MINOCA) has been used as a working diagnosis when criteria for acute MI are fulfilled, coronary angiography demonstrates no obstructive lesion, and no alternative explanation is immediately apparent [2,20]. The Fifth Universal Definition of Myocardial Infarction (2026) introduces the broader terminology of myocardial injury with non-obstructive coronary arteries, emphasizing that non-obstructive angiographic findings do not establish either an ischemic mechanism or a definitive diagnosis of MI [3].

This distinction is clinically important because potential underlying conditions include plaque disruption, coronary embolism, vasospasm, spontaneous coronary artery dissection (SCAD), microvascular dysfunction, myocarditis, Takotsubo syndrome, and other causes of myocardial injury. The diagnostic objective is to determine whether myocardial injury reflects an ischemic infarction, identify the predominant mechanism when possible, and distinguish coronary causes from non-ischemic conditions that require different management [3,20].

#### 4.3.1. Complementary Roles of CMR and Intracoronary Imaging

Cardiovascular magnetic resonance (CMR) characterizes myocardial injury and helps distinguish infarction from myocarditis, Takotsubo syndrome, and other cardiomyopathies [20,21]. Early assessment is preferable when feasible because some diagnostic abnormalities, particularly edema, may become less conspicuous over time.

OCT provides complementary information about occult coronary plaque disruption, thrombus, and selected cases of dissection. In a prospective study of women with MINOCA, OCT identified a definite or possible culprit lesion in 46.2%, CMR was abnormal in 74.1%, and their combination identified a potential cause in 84.5% [21]. Another prospective study including women and men identified an OCT-defined culprit lesion in 45%, while combined OCT and CMR identified a cause in 79% of patients completing both investigations [22].

These findings demonstrate the complementary diagnostic yield of coronary and myocardial imaging. Within the updated diagnostic framework, their principal value is to clarify whether the presentation represents ischemic infarction, non-ischemic myocardial injury, or an alternative cardiac disorder. However, identifying a potential cause does not itself establish that a particular testing sequence or mechanism-directed intervention improves clinical outcomes.

#### 4.3.2. A Hypothesis-Driven Diagnostic Strategy

Evaluation should begin with reassessment of the clinical presentation, serial cardiac troponin findings, electrocardiogram, and coronary angiogram to determine whether the criteria for acute MI are fulfilled and whether an ischemic mechanism is supported [3,4]. This initial assessment should consider overlooked obstructive disease, coronary dissection, supply–demand imbalance, and alternative causes of myocardial injury.

When an occult epicardial mechanism remains suspected, OCT or IVUS may clarify plaque disruption, thrombus, or dissection [16,20,21,22]. Early CMR can help distinguish ischemic myocardial injury from non-ischemic mimics [20,21,22]. If uncertainty persists, coronary function testing may be considered when vasospasm or microvascular dysfunction is suspected [11,13,20]. Coronary computed tomography angiography may provide complementary anatomical information in selected cases.

Test selection should be guided by the unresolved diagnostic question, procedural risk, and likelihood that the result will alter management. This hypothesis-driven strategy is consistent with the emphasis of the Fifth Universal Definition on integrating clinical, biochemical, and imaging findings rather than assigning an infarction diagnosis solely on the basis of troponin elevation or angiographic appearance [3].

### 4.4. From Multimodal Diagnosis to Actionable Precision Phenotyping

The Fifth Universal Definition of Myocardial Infarction provides an updated foundation for distinguishing myocardial injury from infarction and identifying the predominant underlying mechanism [3]. Multimodal assessment should integrate this classification with coronary anatomy, lesion complexity, clinical stability, and patient-specific risk.

Diagnostic findings such as spontaneous coronary artery dissection, non-ischemic myocardial injury, or severe coronary calcification may directly influence management. However, treatment modification based on detailed plaque morphology or other emerging phenotypes requires evidence appropriate to the proposed intervention. The clinical value of additional testing should therefore be assessed according to its ability to resolve diagnostic uncertainty and inform an actionable decision.

## 5. Individualized Revascularization in Acute Coronary Syndromes

### 5.1. Timing of Invasive Management in NSTE-ACS

The timing of invasive management in non–ST-segment elevation acute coronary syndromes (NSTE-ACS) should be individualized according to clinical stability and ischemic risk. Refractory ischemia, hemodynamic or electrical instability, and other very-high-risk features warrant immediate invasive evaluation, whereas clinically stable patients can be stratified according to their anticipated benefit from earlier angiography [1,2].

TIMACS randomized 3031 patients to early or delayed intervention and found no statistically significant difference in the overall primary composite outcome (9.6% vs. 11.3%; hazard ratio [HR], 0.85; *p* = 0.15). However, a prespecified subgroup analysis suggested greater benefit among patients with a GRACE score >140 (HR, 0.65) [23]. These findings support risk-informed invasive timing rather than a uniform time threshold, while recognizing that subgroup evidence does not establish a universally applicable treatment-selection rule.

### 5.2. Vascular Access and Procedural Safety

Vascular access selection illustrates how procedural decisions can be tailored to bleeding risk and anatomical requirements. Randomized evidence generally favors radial access in ACS: RIVAL reduced major vascular complications despite a neutral overall primary composite outcome, while MATRIX demonstrated fewer net adverse clinical events with radial access [24].

Femoral access remains appropriate when radial anatomy is unsuitable or large-bore access and mechanical circulatory support are required. In such patients, ultrasound-guided access and other bleeding-avoidance measures may mitigate procedural risk [1,2]. Thus, access selection should integrate bleeding vulnerability, vascular anatomy, and technical feasibility rather than follow an inflexible procedural preference.

### 5.3. Complete Revascularization in Stable STEMI

Hemodynamic stability is a major determinant of the appropriate extent and timing of revascularization. In stable ST-segment elevation myocardial infarction (STEMI) with multivessel disease, COMPLETE randomized 4041 patients to staged complete revascularization or culprit-only PCI. Cardiovascular death or myocardial infarction occurred in 7.8% versus 10.5%, respectively (HR, 0.74; 95% confidence interval [CI], 0.60–0.91), with the benefit driven predominantly by fewer subsequent myocardial infarctions [25].

The timing of complete revascularization remains a separate decision. COMPLETE supported staged intervention without directly comparing immediate versus staged PCI [25]. MULTISTARS AMI randomized 840 stable STEMI patients and reported a lower one-year primary composite outcome with immediate versus staged complete PCI (8.5% vs. 16.3%), primarily reflecting fewer nonfatal myocardial infarctions and unplanned ischemia-driven revascularizations, without a demonstrated mortality difference [26].

Accordingly, complete revascularization should not be equated with immediate multivessel PCI. Lesion complexity, renal function, contrast exposure, procedural burden, and certainty regarding nonculprit lesions should inform the timing and extent of treatment [1,2].

### 5.4. Revascularization in Cardiogenic Shock

#### 5.4.1. Culprit-Vessel Revascularization During the Acute Phase

Cardiogenic shock illustrates how physiological instability modifies the benefit–harm balance of revascularization. In CULPRIT-SHOCK, 706 patients with acute myocardial infarction, cardiogenic shock, and multivessel disease were randomized to culprit-lesion-only or immediate multivessel PCI. The 30-day composite of death or severe renal failure requiring renal-replacement therapy occurred in 45.9% versus 55.4%, respectively (relative risk, 0.83; 95% CI, 0.71–0.96) [27].

In contrast to stable STEMI, these findings favor limiting initial intervention to the culprit lesion in appropriate patients with shock, highlighting the importance of hemodynamic status in determining revascularization extent [25,27].

#### 5.4.2. Reassessment After Stabilization

Following stabilization, residual coronary anatomy, ventricular function, renal recovery, lesion complexity, and overall prognosis should guide consideration of staged PCI or coronary artery bypass grafting (CABG) [1,2]. When culprit-lesion identification is uncertain, angiographic, electrocardiographic, and ventricular imaging findings may inform the decision, although additional testing should not delay urgent reperfusion [1,2,16].

#### 5.4.3. Mechanical Circulatory Support

Mechanical circulatory support should be considered according to the shock phenotype and anticipated benefit rather than applied routinely. ECLS-SHOCK demonstrated no reduction in 30-day mortality with routine early extracorporeal life support and reported increased bleeding and vascular complications [28].

Conversely, DanGer-Shock reported reduced 180-day mortality with a microaxial-flow pump in a selected STEMI-related shock population, accompanied by increased bleeding, limb ischemia, hemolysis, and other device-related complications [29]. Because these trials evaluated different devices and populations, their findings do not establish a universally preferred support strategy. Patient selection should account for ventricular failure pattern, shock severity, reversibility, vascular anatomy, and device-related risks [28,29].

### 5.5. Lesion-Specific and Imaging-Guided PCI

Lesion complexity influences procedural strategy, particularly in calcified, bifurcation, and left-main disease. Intracoronary imaging can refine lesion assessment, guide device selection and stent optimization, and identify abnormalities such as underexpansion or edge dissection that may be underestimated by angiography [16].

Randomized evidence from RENOVATE-COMPLEX-PCI and OCTOBER supports imaging-guided PCI in selected complex lesions [17,18]. The technical characteristics of optical coherence tomography (OCT) and intravascular ultrasound (IVUS) are discussed in Section 4.2. In clinical practice, modality selection should reflect coronary anatomy, contrast considerations, procedural objectives, and operator expertise. Imaging guidance is most clinically meaningful when it informs a defined procedural decision rather than serving as additional characterization alone.

### 5.6. Precision Revascularization: Integrating Anatomy, Physiology, and Patient Risk

Precision revascularization requires integration of coronary anatomy, hemodynamic status, lesion complexity, ventricular function, comorbidity, and procedural risk [1,2]. When the choice between PCI and CABG is uncertain, anticipated completeness of revascularization, surgical risk, patient preferences, and Heart Team assessment should inform treatment selection.

Patient vulnerability further modifies the expected balance of benefit and harm. Chronic kidney disease, anemia, frailty, previous bleeding, and multimorbidity may increase procedural risk, whereas recurrent ischemia, ventricular dysfunction, and high-risk anatomy may increase potential benefit. Functional status, life expectancy, and patient goals should complement chronological age.

Ultimately, individualized revascularization concerns the timing, extent, modality, and procedural intensity of intervention, with reassessment as clinical status evolves. The strongest examples of clinically actionable precision are those in which patient characteristics directly inform treatment decisions supported by randomized clinical-outcome evidence.

## 6. Personalized Antithrombotic Therapy in Acute Coronary Syndromes

### 6.1. From Standard DAPT to Dynamic Antithrombotic Therapy

Dual antiplatelet therapy (DAPT) with aspirin and a P2Y12 inhibitor remains the default after ACS, generally for 12 months in patients without high bleeding risk, with prasugrel or ticagrelor preferred over clopidogrel in appropriate patients [1,2]. However, treatment intensity and duration should reflect the evolving balance between ischemic protection and bleeding harm.

Precision antithrombotic strategies include abbreviated DAPT followed by P2Y12-inhibitor monotherapy, de-escalation of P2Y12 inhibition, extended antithrombotic therapy, and modified regimens for patients requiring oral anticoagulation. These approaches address different clinical decisions and should be selected according to patient characteristics, treatment timing, and the populations in which benefit has been demonstrated.

### 6.2. Assessing Ischemic and Bleeding Risk

Ischemic and bleeding risks frequently overlap, particularly in patients with advanced age, chronic kidney disease, anemia, diabetes, or extensive coronary disease. Consequently, individualized antithrombotic treatment requires simultaneous assessment of both risks rather than reliance on isolated characteristics.

The Academic Research Consortium for High Bleeding Risk (ARC-HBR) identifies high bleeding risk through at least one major or two minor criteria [30]. PRECISE-DAPT incorporates age, creatinine clearance, hemoglobin, white blood cell count, and previous bleeding, with a score ≥25 indicating high bleeding risk [31]. Neither tool independently determines the optimal antiplatelet regimen.

Ischemic risk assessment should incorporate previous myocardial infarction or stent thrombosis, diabetes, chronic kidney disease, peripheral arterial disease, ventricular dysfunction, multivessel disease, incomplete revascularization, and complex PCI [1,2]. The DAPT score addresses a distinct decision: whether prolonged DAPT may be appropriate in selected patients who have completed an initial event-free treatment period [32].

These tools should be applied within their validated clinical contexts, with reassessment following recurrent ischemia, bleeding, renal deterioration, or new indications for anticoagulation.

### 6.3. Abbreviated DAPT and P2Y12-Inhibitor Monotherapy

Aspirin withdrawal after an initial DAPT period may reduce bleeding in selected patients, although treatment effects depend on the timing of withdrawal, the P2Y12 inhibitor continued, and trial eligibility.

TWILIGHT randomized 7119 high-risk PCI patients who remained event-free after three months of ticagrelor-based DAPT. Ticagrelor monotherapy reduced BARC type 2, 3, or 5 bleeding compared with continued DAPT (4.0% vs. 7.1%; hazard ratio [HR], 0.56), without an observed increase in death, myocardial infarction, or stroke [33]. However, the study was not restricted to ACS, and its run-in design limits generalizability to patients experiencing early complications.

In selected ACS populations, TICO and ULTIMATE-DAPT supported ticagrelor monotherapy after three months and one month of DAPT, respectively, with reductions in bleeding and no clear increase in major ischemic events [34,35]. MASTER DAPT supported abbreviated therapy in selected high-bleeding-risk PCI patients, although its population and subsequent treatment regimens were heterogeneous [36].

Importantly, STOPDAPT-2 ACS did not establish noninferiority for one to two months of DAPT followed predominantly by clopidogrel monotherapy [37]. Thus, short-DAPT strategies should not be treated as interchangeable; clinical application must reflect the specific regimen, transition timing, and population studied.

### 6.4. De-Escalation of P2Y12 Inhibition

De-escalation reduces P2Y12-inhibitor intensity rather than withdrawing aspirin and may be considered when bleeding susceptibility or treatment tolerance favors a less potent regimen.

TROPICAL-ACS demonstrated noninferior net clinical benefit with platelet-function-guided de-escalation from prasugrel to clopidogrel, incorporating escalation when high platelet reactivity was identified [38]. POPular Genetics demonstrated noninferior thrombotic outcomes with less bleeding using CYP2C19 genotype-guided P2Y12-inhibitor selection after primary PCI [39]. These trials provide examples of biomarker- or test-guided treatment selection supported by randomized clinical evidence.

Unguided strategies have also been investigated. TALOS-AMI reported fewer net adverse events, predominantly through reduced bleeding, after switching stabilized patients from ticagrelor-based to clopidogrel-based DAPT following one month of treatment [40]. HOST-REDUCE-POLYTECH-ACS evaluated prasugrel dose reduction rather than drug substitution [41].

Guided switching, unguided switching, and dose reduction represent distinct strategies. Their application should reflect trial eligibility, timing, bleeding susceptibility, and ischemic risk, with particular caution in patients with recurrent ischemia, previous stent thrombosis, or complex PCI [1,2,38,39,40,41].

### 6.5. Extended Antithrombotic Therapy and Residual Thrombotic Risk

Patients with recurrent ischemic events, multivessel disease, diabetes, chronic kidney disease, or peripheral arterial disease may retain substantial atherothrombotic risk beyond the first year after myocardial infarction [1,2]. However, treatment intensification requires evidence that the expected ischemic benefit outweighs bleeding harm.

PEGASUS-TIMI 54 randomized 21,162 patients with prior myocardial infarction and additional risk factors. Ticagrelor 60 mg twice daily plus aspirin reduced cardiovascular death, myocardial infarction, or stroke compared with aspirin alone (7.77% vs. 9.04%; HR, 0.84), while increasing TIMI major bleeding (2.30% vs. 1.06%) [42].

COMPASS evaluated a distinct strategy in stable coronary or peripheral atherosclerotic disease. Rivaroxaban 2.5 mg twice daily plus aspirin reduced cardiovascular death, stroke, or myocardial infarction compared with aspirin alone (4.1% vs. 5.4%; HR, 0.76), but increased major bleeding (3.1% vs. 1.9%; HR, 1.70) [43].

These findings support selective long-term intensification in appropriately eligible patients rather than routine extension of antithrombotic therapy after ACS. Importantly, the COMPASS regimen should not be interpreted as a substitute for standard early post-ACS DAPT.

### 6.6. ACS in Patients Requiring Oral Anticoagulation

Concomitant oral anticoagulation introduces competing risks of systemic thromboembolism, coronary thrombosis, and bleeding. In patients with atrial fibrillation undergoing PCI, PIONEER AF-PCI, RE-DUAL PCI, AUGUSTUS, and ENTRUST-AF PCI generally demonstrated less bleeding with strategies limiting aspirin exposure and combining oral anticoagulation with a P2Y12 inhibitor than with prolonged vitamin K antagonist-based triple therapy [44,45,46,47]. AUGUSTUS additionally demonstrated less bleeding with apixaban than with a vitamin K antagonist and greater bleeding with aspirin than with placebo [46].

Contemporary guidance generally supports a short initial period of triple therapy followed by oral anticoagulation plus a P2Y12 inhibitor, usually clopidogrel, with treatment duration individualized according to stent-thrombosis and bleeding risk [1,2]. Direct oral anticoagulants are generally preferred in eligible patients, while vitamin K antagonists remain necessary for selected indications, including mechanical prosthetic heart valves.

These findings should be applied according to the anticoagulation indication and trial population rather than generalized to all patients requiring anticoagulation. Further discussion of atrial fibrillation management during long-term secondary prevention is provided in Section 7.6.

### 6.7. Integrated Precision Antithrombotic Strategy

Precision antithrombotic therapy requires repeated evaluation of ischemic risk, bleeding susceptibility, procedural characteristics, and treatment response. Following the initial high-risk period, selected patients may benefit from abbreviated DAPT, aspirin withdrawal, or P2Y12-inhibitor de-escalation [33,34,35,36,37,38,39,40,41], while patients requiring anticoagulation need regimens that minimize unnecessary combination therapy [44,45,46,47].

Long-term treatment intensification should be reserved for appropriately selected patients with persistent atherothrombotic risk and acceptable bleeding susceptibility [42,43]. The clinically actionable principle is to match drug combination, potency, and duration to patient-specific benefit–harm considerations, with reassessment as clinical circumstances evolve.

## 7. Residual Biological Risk and Precision Secondary Prevention

### 7.1. Residual Biological Risk After ACS

Despite successful treatment of the acute event, patients remain susceptible to recurrent cardiovascular events because of persistent atherogenic lipoprotein exposure, inflammation, thrombotic susceptibility, cardiometabolic disease, and atherosclerosis beyond the culprit lesion [1,2]. These pathways frequently coexist, but identifying multiple abnormalities does not necessarily justify multiple additional treatments.

Precision secondary prevention requires optimization of established therapies followed by identification of residual risks that may support further evidence-based intervention. Persistent low-density lipoprotein cholesterol (LDL-C) elevation, elevated lipoprotein(a) [Lp(a)], inflammatory activity, diabetes, obesity, chronic kidney disease (CKD), and heart failure may influence subsequent management. Residual thrombotic risk and bleeding susceptibility are addressed in Section 6.

The priority is to match additional treatment to patient characteristics and demonstrated clinical benefit rather than to the number of identifiable biological abnormalities.

### 7.2. Early Intensive LDL-C Lowering

#### 7.2.1. Early Treatment and LDL-C Targets

High-intensity statin therapy should be initiated or intensified early after ACS unless contraindicated or not tolerated [1,2]. The 2023 ESC guidelines recommend LDL-C below 55 mg/dL (1.4 mmol/L) and a reduction of at least 50% from baseline [2]. U.S. ACS guidance recommends adding nonstatin therapy when LDL-C remains ≥ 70 mg/dL despite maximally tolerated statin treatment and considers intensification reasonable at 55–69 mg/dL [1]. The 2026 ACC/AHA dyslipidemia guideline specifies LDL-C goals below 55 mg/dL for very-high-risk atherosclerotic cardiovascular disease (ASCVD) and below 70 mg/dL for ASCVD not classified as very high risk [48].

IMPROVE-IT demonstrated that adding ezetimibe to simvastatin reduced cardiovascular events after ACS [49]. Baseline LDL-C, previous treatment, and the reduction required to reach the intended goal should inform whether early combination therapy is appropriate.

#### 7.2.2. PCSK9 Inhibition and Cardiovascular Outcomes

Proprotein convertase subtilisin/kexin type 9 (PCSK9) monoclonal antibodies provide additional LDL-C reduction with demonstrated cardiovascular-outcome benefit. FOURIER established benefit with evolocumab in stable ASCVD [50], while ODYSSEY OUTCOMES demonstrated benefit with alirocumab following recent ACS [51].

Treatment selection should reflect baseline LDL-C, previous therapy, additional reduction required, tolerability, adherence, and access. Importantly, evidence supporting PCSK9 inhibition for cardiovascular-event reduction should be distinguished from studies assessing the effects of very early treatment on lipid or plaque-related endpoints.

#### 7.2.3. Very Early PCSK9 Inhibition After ACS

EVOPACS randomized 308 hospitalized ACS patients and demonstrated rapid LDL-C lowering when evolocumab was initiated during hospitalization alongside atorvastatin [52]. PACMAN-AMI evaluated 300 patients with acute myocardial infarction and found that early alirocumab treatment improved nonculprit plaque characteristics, including atheroma regression, lipid burden, and fibrous-cap thickness, over 52 weeks [53].

These findings support the feasibility and biological effects of very early PCSK9 inhibition but do not establish that initiation during ACS hospitalization improves clinical outcomes. EVOPACS primarily assessed LDL-C lowering, whereas PACMAN-AMI assessed intracoronary imaging endpoints. Their results should therefore be interpreted alongside, rather than as substitutes for, the clinical-outcome evidence from FOURIER and ODYSSEY OUTCOMES [50,51,52,53].

#### 7.2.4. From Sequential Escalation to Early Combination Therapy

The index ACS hospitalization provides an opportunity to identify patients unlikely to achieve the intended LDL-C reduction with statin monotherapy. Early combination treatment may be appropriate when baseline LDL-C is markedly elevated, previous treatment has been insufficient, or substantial additional lowering is required [1,2,49,51].

Postdischarge LDL-C reassessment should guide treatment intensification, adherence evaluation, and regimen adjustment. This approach combines early intervention with response-guided management rather than prolonged sequential escalation [1,2].

### 7.3. Bempedoic Acid, Inclisiran, and Emerging Lipid-Targeted Strategies

#### 7.3.1. Bempedoic Acid

Bempedoic acid is an oral ATP-citrate lyase inhibitor that provides an additional LDL-C-lowering option, particularly for patients with statin intolerance or an inadequate response to tolerated therapy.

CLEAR Outcomes randomized 13,970 statin-intolerant patients, approximately 70% of whom had established cardiovascular disease. Over a median follow-up of 40.6 months, bempedoic acid reduced cardiovascular death, nonfatal myocardial infarction, nonfatal stroke, or coronary revascularization compared with placebo (11.7% vs. 13.3%; hazard ratio [HR], 0.87; 95% confidence interval [CI], 0.79–0.96). Significant reductions in cardiovascular or all-cause mortality were not demonstrated [54].

Bempedoic acid therefore has cardiovascular-outcome evidence in a relevant but not exclusively recent-ACS population. Selection should consider statin tolerance, additional LDL-C reduction required, and adverse effects, including hyperuricemia, gout, and cholelithiasis [54].

#### 7.3.2. Inclisiran

Inclisiran is a small interfering RNA therapy that reduces hepatic PCSK9 synthesis and provides sustained LDL-C lowering with infrequent maintenance dosing. ORION-10 and ORION-11 demonstrated substantial LDL-C reductions in patients receiving background lipid-lowering therapy [55].

Its dosing schedule may offer practical advantages for selected patients requiring additional LDL-C lowering. However, the LDL-C findings from these trials should not be equated with demonstrated cardiovascular-event reduction. Clinical-outcome evidence must be evaluated separately from lipid-lowering efficacy [50,51,55].

#### 7.3.3. Lipoprotein(a) and Emerging Targeted Therapies

Lipoprotein(a) [Lp(a)] is a largely genetically determined contributor to residual atherosclerotic cardiovascular risk. Measurement may refine risk assessment, particularly in patients with premature or recurrent atherosclerotic cardiovascular disease or a strong family history.

RNA-based therapies can substantially reduce circulating Lp(a), but reductions in this biomarker should not be interpreted as proof of cardiovascular benefit. The Lp(a)HORIZON trial evaluated pelacarsen in 8323 patients with established cardiovascular disease and elevated Lp(a) [56]. A September 2026 company announcement reported that the trial did not meet its primary cardiovascular endpoint; full peer-reviewed outcome results were unavailable at manuscript preparation [57].

Because this information derives from a preliminary corporate communication rather than a peer-reviewed trial report, detailed interpretation of treatment effects is not warranted. The clinical role of Lp(a)-targeted therapies should be determined by definitive cardiovascular-outcome evidence.

#### 7.3.4. Matching Lipid-Lowering Therapy to the Evidence

Statins, ezetimibe, PCSK9 monoclonal antibodies, and bempedoic acid have cardiovascular-outcome evidence, although the applicability of individual trials to recent ACS differs. Inclisiran provides substantial LDL-C lowering, while emerging Lp(a)-targeted strategies require further outcome-based evaluation [49,50,51,52,54,55,56,57].

Treatment selection should integrate baseline LDL-C, previous therapy, statin tolerance, adherence, comorbidities, and the applicability of trial evidence. The objective is sustained lipid lowering with a clinically appropriate regimen rather than treatment selection based solely on biomarker reduction.

### 7.4. Residual Inflammatory Risk and Anti-Inflammatory Therapy

#### 7.4.1. Residual Inflammation as a Therapeutic Target

Inflammatory activity may persist despite intensive lipid lowering and contribute to recurrent atherosclerotic events. High-sensitivity C-reactive protein (hs-CRP) provides prognostic information but is not specific to vascular inflammation and does not independently establish treatment benefit [58].

CANTOS randomized 10,061 patients with previous myocardial infarction and hs-CRP ≥ 2 mg/L. Canakinumab reduced recurrent cardiovascular events without materially lowering LDL-C, demonstrating the therapeutic relevance of inflammatory pathways. However, serious infections increased, and all-cause mortality was not reduced [58]. These findings do not support routine canakinumab use after ACS.

#### 7.4.2. Colchicine Across Different Phases of Coronary Disease

COLCOT randomized 4745 patients within 30 days after myocardial infarction. Colchicine 0.5 mg daily reduced the composite of cardiovascular death, resuscitated cardiac arrest, myocardial infarction, stroke, or urgent hospitalization for angina leading to revascularization compared with placebo (5.5% vs. 7.1%; HR, 0.77; 95% CI, 0.61–0.96), primarily through fewer strokes and urgent revascularizations [59].

LoDoCo2 subsequently demonstrated cardiovascular benefit in chronic coronary disease, reducing its primary composite outcome from 9.6% to 6.8% (HR, 0.69; 95% CI, 0.57–0.83) [60]. However, evidence from chronic coronary disease should not be directly extrapolated to routine treatment initiation during acute myocardial infarction.

#### 7.4.3. CLEAR SYNERGY and Heterogeneity of Clinical Evidence

CLEAR SYNERGY evaluated colchicine initiated soon after myocardial infarction in more than 7000 patients undergoing PCI. Over approximately three years, the primary composite cardiovascular outcome occurred in 9.1% of colchicine-treated patients and 9.3% of placebo-treated patients (HR, 0.99; 95% CI, 0.85–1.16), despite a reduction in C-reactive protein [61].

The differing findings of COLCOT, LoDoCo2, and CLEAR SYNERGY highlight uncertainty regarding the generalizability of colchicine benefit across coronary disease populations. Differences in patient selection, treatment timing, background therapy, and endpoint composition may contribute, but these explanations remain unconfirmed [59,60,61].

#### 7.4.4. Clinical Integration

The 2025 ACC/AHA ACS guideline states that low-dose colchicine may be reasonable after ACS to reduce major adverse cardiovascular events (Class IIb, Level B-R) [1]. Given the heterogeneous trial findings, treatment should be considered selectively according to residual atherosclerotic risk, tolerability, renal and hepatic function, and drug interactions.

Persistent hs-CRP elevation may inform risk assessment, but routine hs-CRP-guided colchicine selection has not been established. Anti-inflammatory treatment should complement, rather than replace, established secondary prevention [58,59,60,61].

### 7.5. Cardiometabolic Risk: Diabetes, Obesity, SGLT2 Inhibitors, and GLP-1 Receptor Agonists

#### 7.5.1. Cardiometabolic Phenotyping After ACS

Diabetes, obesity, CKD, and heart failure contribute to residual cardiovascular risk after ACS and may establish indications for treatments with cardiovascular or organ-protective benefits [1,2]. Sodium–glucose cotransporter-2 (SGLT2) inhibitors and glucagon-like peptide-1 receptor agonists (GLP-1 RAs) should be considered according to patient characteristics and the populations in which individual therapies have demonstrated benefit [62,63,64].

#### 7.5.2. SGLT2 Inhibitors After Myocardial Infarction

SGLT2 inhibitors have established cardiovascular and renal benefits in appropriate patients with heart failure, CKD, or type 2 diabetes. However, routine initiation solely because of an acute myocardial infarction requires separate evidence.

EMPACT-MI randomized 6522 patients with acute myocardial infarction and increased heart-failure risk. Empagliflozin did not significantly reduce the primary composite of first hospitalization for heart failure or all-cause death (8.2% vs. 9.1%; HR, 0.90; 95% CI, 0.76–1.06; *p* = 0.21), although first heart-failure hospitalization was lower (3.6% vs. 4.7%; HR, 0.77; 95% CI, 0.60–0.98) [62].

DAPA-MI evaluated dapagliflozin after myocardial infarction in patients without established diabetes or chronic heart failure. Improvements in its hierarchical cardiometabolic composite were driven predominantly by metabolic outcomes, without establishing a reduction in conventional hard cardiovascular events [63].

Accordingly, SGLT2 inhibitor use after ACS should follow established heart-failure, renal, or diabetes-related indications rather than routine prescription based on myocardial infarction alone.

#### 7.5.3. GLP-1 Receptor Agonists, Diabetes, and Obesity

Selected GLP-1 RAs reduce major cardiovascular events in patients with type 2 diabetes and established or high-risk ASCVD, although treatment effects should be assessed at the individual-agent level.

SELECT randomized 17,604 patients with established cardiovascular disease and overweight or obesity, but without diabetes. Semaglutide 2.4 mg weekly reduced cardiovascular death, nonfatal myocardial infarction, or nonfatal stroke compared with placebo (6.5% vs. 8.0%; HR, 0.80; 95% CI, 0.72–0.90) [64].

These findings support semaglutide for long-term cardiovascular risk reduction in eligible patients but do not establish a benefit from routine initiation during acute ACS hospitalization. Lifestyle modification, cardiac rehabilitation, and weight management remain essential components of care [1,2,64].

#### 7.5.4. Matching Therapy to the Cardiometabolic Phenotype

Cardiometabolic treatment should follow established indications and relevant clinical-outcome evidence. SGLT2 inhibitors may be appropriate when heart failure, CKD, or diabetes-related indications are present, while GLP-1 RAs with demonstrated cardiovascular benefit may be appropriate for eligible patients with type 2 diabetes or overweight or obesity and established cardiovascular disease [1,2,64].

The distinction between chronic-disease treatment and routine acute post-myocardial-infarction initiation remains important. EMPACT-MI did not significantly reduce its primary endpoint with early empagliflozin treatment [62], whereas SELECT demonstrated cardiovascular benefit with semaglutide in an eligible chronic cardiovascular disease population [64].

### 7.6. Guideline-Directed Management of Cardiovascular Comorbidities Following ACS

Long-term management after acute coronary syndrome (ACS) requires comprehensive treatment of cardiovascular comorbidities in addition to coronary revascularization and antithrombotic therapy. Atrial fibrillation (AF), diabetes mellitus, dyslipidemia, and hypertension contribute to recurrent cardiovascular events and may influence treatment selection, medication safety, and long-term prognosis. Contemporary clinical guidelines emphasize individualized secondary prevention based on thromboembolic and bleeding risk, atherosclerotic disease burden, ventricular function, kidney function, metabolic characteristics, and treatment tolerance [1,2]. Accordingly, precision management after ACS should integrate disease-specific recommendations with longitudinal reassessment of cardiovascular risk.

#### 7.6.1. Atrial Fibrillation

AF complicates post-ACS management because patients may require oral anticoagulation for prevention of cardioembolic stroke while simultaneously receiving antiplatelet therapy for coronary protection. Stroke prevention should be guided by validated thromboembolic risk assessment, with direct oral anticoagulants generally preferred over vitamin K antagonists in eligible patients, excluding those with mechanical heart valves or moderate-to-severe mitral stenosis [65]. In patients with AF undergoing percutaneous coronary intervention (PCI), contemporary European guidelines generally favor a short initial period of triple antithrombotic therapy, followed by oral anticoagulation combined with a single antiplatelet agent, usually clopidogrel. Triple therapy is commonly limited to approximately one week, although extension up to one month may be considered when stent-thrombosis risk outweighs bleeding risk [2,65]. Following the initial combination-treatment period, oral anticoagulant monotherapy is generally preferred in clinically stable patients beyond 12 months without another indication for antiplatelet therapy [65]. Treatment decisions should incorporate renal function, bleeding history, procedural complexity, and recurrent ischemic events. Rate or rhythm control should be individualized according to symptoms, ventricular function, AF characteristics, and patient preferences. These considerations are consistent with the 2024 European Society of Cardiology guidelines for AF management [65].

#### 7.6.2. Diabetes Mellitus

Diabetes mellitus is an important determinant of residual cardiovascular risk following ACS and requires management extending beyond glycemic control alone. The American Diabetes Association Standards of Care in Diabetes (2026) recommend sodium–glucose cotransporter-2 (SGLT2) inhibitors and/or glucagon-like peptide-1 receptor agonists (GLP-1 RAs) with demonstrated cardiovascular benefit for patients with type 2 diabetes and established atherosclerotic cardiovascular disease, with treatment selection guided by concomitant heart failure, chronic kidney disease, obesity, and individual clinical characteristics. These therapies may provide cardiovascular or renal benefits independently of their glucose-lowering effects [66]. Glycemic targets should be individualized according to age, frailty, hypoglycemia susceptibility, comorbidity burden, and life expectancy; an HbA1c target below 7% is appropriate for many nonpregnant adults, although less stringent targets may be warranted in selected patients [67]. Following ACS, treatment should also address smoking cessation, physical activity, dietary modification, weight management, renal protection, and medication adherence [1,2,66]. Importantly, the cardiovascular benefits established in populations with type 2 diabetes and atherosclerotic disease should not be interpreted as evidence supporting routine initiation of these agents in all patients solely because an acute myocardial infarction has occurred [62,63].

#### 7.6.3. Dyslipidemia

Intensive lipid lowering is a central component of secondary prevention following ACS. High-intensity statin therapy should be initiated or continued unless contraindicated, with additional lipid-lowering treatment selected according to baseline low-density lipoprotein cholesterol (LDL-C), achieved reduction, residual cardiovascular risk, and treatment tolerance. The 2025 American College of Cardiology/American Heart Association ACS guideline recommends adding nonstatin lipid-lowering therapy when LDL-C remains at or above 70 mg/dL despite maximally tolerated statin therapy and considers further intensification reasonable at LDL-C concentrations of 55–69 mg/dL [1]. The 2025 European Society of Cardiology/European Atherosclerosis Society focused update maintains the established LDL-C goal below 55 mg/dL, together with a reduction of at least 50% from baseline, for patients at very high cardiovascular risk [68]. The 2026 American dyslipidemia guideline further emphasizes risk-based LDL-C goals in secondary prevention [48]. Ezetimibe and proprotein convertase subtilisin/kexin type 9 monoclonal antibodies are established options for additional LDL-C reduction, while other nonstatin agents may be considered according to guideline recommendations, patient characteristics, and available outcome evidence [48,49,50,51,52,54]. Lipid measurements should be reassessed approximately four to eight weeks after initiation or modification of lipid-lowering treatment to evaluate treatment response and guide intensification [1]. Assessment of lipoprotein(a), when appropriate, may further refine residual cardiovascular risk, although elevated concentrations alone do not establish the benefit of an unproven targeted intervention [48,56,57].

#### 7.6.4. Hypertension

Blood pressure control is essential for reducing recurrent cardiovascular events after ACS. The 2025 American College of Cardiology/American Heart Association hypertension guideline recommends a general treatment goal below 130/80 mmHg, with individualization according to clinical characteristics and tolerability [69]. The 2024 European Society of Cardiology guideline recommends targeting a systolic blood pressure of 120–129 mmHg in most adults receiving antihypertensive therapy, provided treatment is well tolerated, with less intensive targets considered for patients with symptomatic orthostatic hypotension, significant frailty, advanced age, or limited life expectancy [70]. Following myocardial infarction, antihypertensive treatment should also account for ventricular systolic function, heart failure, persistent angina, kidney function, and other indications for specific pharmacological agents. Beta-blockers and renin–angiotensin system inhibitors may be particularly relevant when supported by post-infarction clinical indications, while calcium-channel blockers and thiazide or thiazide-like diuretics may be used when additional blood pressure reduction is required [1,69,70]. Treatment should avoid symptomatic hypotension and excessive reductions in diastolic pressure, particularly in patients with significant coronary artery disease. Home blood pressure monitoring, medication adherence assessment, and periodic evaluation of renal function and electrolytes support safe and sustained blood pressure control [69,70].

#### 7.6.5. Integration into Longitudinal Precision Secondary Prevention

The management of cardiovascular comorbidities following ACS requires an integrated, individualized approach. Atrial fibrillation, diabetes, dyslipidemia, and hypertension influence treatment selection, cardiovascular risk, and medication safety. Regular reassessment of clinical status, treatment response, and competing ischemic and bleeding risks is therefore essential to optimize long-term secondary prevention [1,2,65,66,68,69,70].

### 7.7. Integrated Precision Secondary Prevention

Precision secondary prevention should begin with optimization of established treatments, medication adherence, risk-factor control, and cardiac rehabilitation [1,2]. Additional interventions should address residual risks for which clinically relevant treatment benefit has been demonstrated.

Persistent LDL-C elevation may justify intensified lipid lowering [49,50,51,52,54,55], while selected patients may be considered for anti-inflammatory therapy [58,59,60,61] or cardiometabolic treatments according to established indications [62,63,64]. Elevated Lp(a) may refine risk assessment, but biomarker reduction alone does not establish clinical benefit [56,57].

Treatment priorities should be reassessed as cardiovascular risk, ventricular function, comorbidities, treatment response, and patient preferences evolve. Table 1 summarizes the relationship between the four precision-medicine domains, clinical decisions, and longitudinal reassessment.

## 8. Future Directions in Precision ACS Care

Future research in precision medicine for ACS should move beyond increasingly detailed patient characterization toward demonstrating that phenotype-guided strategies improve treatment selection and clinical outcomes. Advances in artificial intelligence (AI), multimodal imaging, molecular profiling, and longitudinal risk prediction offer opportunities to identify clinically relevant heterogeneity. However, successful translation requires prospective validation, evidence of differential treatment benefit, and integration into routine clinical workflows. Research priorities should therefore focus on determining which patient characteristics can reliably inform therapeutic decisions, when those decisions should be reassessed, and whether the resulting strategies improve outcomes compared with established guideline-directed care.

### 8.1. Multimodal Data Integration and Artificial Intelligence

AI-based approaches may facilitate integration of clinical characteristics, electrocardiographic findings, high-sensitivity cardiac troponin trajectories, coronary anatomy, intracoronary imaging, cardiovascular magnetic resonance (CMR), and longitudinal clinical data to support diagnosis, risk stratification, and treatment planning [71]. By analyzing interactions among variables that are difficult to capture through conventional risk scores, machine-learning models may identify patterns associated with recurrent ischemic events, bleeding, ventricular dysfunction, or other clinically important outcomes.

Automated analysis of optical coherence tomography (OCT) and intravascular ultrasound (IVUS) may improve the reproducibility and efficiency of plaque characterization, vessel measurements, and stent assessment [16,71]. Integration of imaging findings with clinical and laboratory data may also support more comprehensive identification of anatomical and biological risk profiles. However, improved image classification or predictive discrimination does not necessarily establish that AI-assisted management improves patient outcomes.

Future investigations should distinguish models developed for diagnosis or prognosis from those intended to guide treatment selection. The latter require evaluation of whether model-informed decisions improve the expected balance of benefit and harm compared with conventional clinical assessment. Prospective studies should assess calibration, discrimination, clinical utility, and the consequences of incorrect recommendations, ideally followed by randomized evaluations of AI-assisted decision pathways.

Important implementation challenges include incomplete or inconsistent clinical data, variation in imaging protocols, differences between healthcare systems, algorithmic bias, and model performance deterioration over time. External validation across diverse populations, interpretability, data privacy, clinician oversight, and integration into existing workflows are essential before routine clinical implementation.

### 8.2. Molecular Endotyping and Biomarker-Guided Therapy

Advances in genomics, transcriptomics, proteomics, metabolomics, and other molecular profiling techniques may help characterize biological heterogeneity among patients with ACS. Integrated multi-omic approaches could identify distinct inflammatory, thrombotic, lipid-related, or metabolic endotypes that are not fully captured by conventional clinical characteristics or circulating biomarker concentrations.

Such endotypes may improve understanding of disease mechanisms and residual cardiovascular risk. However, their therapeutic relevance depends on whether they identify patients who respond differently to specific interventions. Associations between molecular signatures and adverse outcomes establish prognostic information but do not necessarily demonstrate that treatment selection based on those signatures improves clinical outcomes.

Inflammatory biomarkers, including high-sensitivity C-reactive protein, may help characterize residual inflammatory risk, while lipoprotein(a) [Lp(a)] contributes information about genetically influenced atherosclerotic risk [56,57,58,59,60,61]. Nevertheless, biomarker concentrations should not be assumed to predict treatment benefit solely because they are associated with cardiovascular events. Prospective investigations should determine whether predefined biomarker thresholds or molecular signatures identify differential benefit or harm from anti-inflammatory, lipid-lowering, antithrombotic, or cardiometabolic therapies.

Future biomarker-guided trials should prospectively specify the biomarker or molecular signature, analytical method, eligibility threshold, treatment strategy, comparator, and clinically meaningful endpoint. Randomized comparisons should assess treatment-by-biomarker interactions where appropriate, rather than relying exclusively on within-subgroup statistical significance. Reproducibility, assay standardization, affordability, and accessibility will also influence whether molecular endotyping can be translated into routine clinical practice.

### 8.3. From Plaque Phenotyping to Mechanism-Guided Treatment

Intracoronary imaging has improved the identification of plaque rupture, plaque erosion, calcified nodules, and other mechanisms of coronary instability [7,8,9,16]. Emerging causal endotype–based approaches to myocardial infarction classification may further distinguish clinically heterogeneous populations according to the predominant underlying mechanism [14]. These advances provide a foundation for more targeted investigation, but prospective treatment studies are needed to determine which mechanistic distinctions have actionable therapeutic implications.

One important research priority is the evaluation of stent-avoidance strategies in carefully selected patients with OCT-defined plaque erosion. Existing proof-of-concept evidence supports further investigation, but randomized clinical-outcome trials are needed to establish whether intensive antithrombotic therapy without immediate stenting provides comparable or superior outcomes to conventional intervention [9].

Similarly, research should examine whether detailed characterization of coronary calcification can improve the selection of lesion-preparation techniques or other procedural strategies beyond established anatomical and procedural criteria. Such studies should evaluate clinically meaningful endpoints rather than relying solely on improvements in stent expansion or imaging-derived measurements.

Mechanism-directed management also warrants investigation in patients with non-obstructive coronary arteries, coronary vasospasm, and coronary microvascular dysfunction [11,13,20,21,22]. In these populations, improved diagnostic classification may help identify distinct therapeutic targets, but the benefits of testing-guided interventions should be established through appropriately designed comparative studies.

Future research should also evaluate whether combining culprit-lesion characteristics with total coronary atherosclerotic burden, nonculprit plaque features, and systemic biological markers can identify patients who derive additional benefit from intensified secondary prevention. This approach may help connect local coronary mechanisms with the broader determinants of recurrent cardiovascular risk.

### 8.4. Dynamic Risk Prediction and Clinical Implementation

Precision ACS management should account for the fact that patient characteristics and treatment priorities change over time. Ischemic and bleeding risks may evolve following revascularization, while renal function, hemoglobin concentration, ventricular function, lipid response, medication adherence, and the development of new comorbidities can alter the expected benefits and harms of treatment [1,2].

Dynamic prediction models may integrate repeated clinical assessments, laboratory measurements, imaging findings, and treatment responses to update individualized risk estimates after hospital discharge. Such models could potentially support decisions regarding antithrombotic intensity and duration, lipid-lowering escalation, cardiometabolic therapy, and the need for further investigation.

However, dynamic risk prediction should be distinguished from dynamic treatment optimization. A model that accurately predicts recurrent cardiovascular events or bleeding does not necessarily identify which treatment adjustment will improve outcomes. Future studies should therefore determine whether repeated model-guided reassessment improves clinical decisions compared with established guideline-directed follow-up.

Potential research designs include prospective validation cohorts, randomized trials of model-guided versus conventional management, and adaptive treatment studies evaluating predefined reassessment intervals and treatment-modification rules. Evaluation should incorporate clinical outcomes, calibration over time, treatment burden, patient preferences, and the consequences of false-positive or false-negative risk predictions.

Implementation research should also address interoperability between electronic health records, imaging platforms, and laboratory systems; clinician workload; transparency of recommendations; and the cost-effectiveness of advanced diagnostic strategies. Particular attention is needed to ensure that sophisticated risk models and molecular technologies do not widen disparities in cardiovascular care. Representative study populations and evaluation across different healthcare settings are essential to establish generalizability and equitable clinical utility.

### 8.5. Research Priorities

The central research priority is to move from describing ACS heterogeneity toward prospectively testing interventions that act on clinically meaningful differences between patients. Promising areas include mechanism-guided treatment of coronary instability, AI-assisted decision support, imaging-informed secondary prevention, biomarker-stratified anti-inflammatory therapy, Lp(a)-targeted interventions, molecular endotype–guided treatment, and longitudinal models that support adaptive therapeutic decisions [7,8,9,16,56,57,58,59,60,61,71].

Future studies should distinguish three sequential objectives: establishing reproducible diagnostic or biological phenotypes, determining whether these phenotypes identify differences in prognosis or treatment response, and demonstrating that phenotype-guided management improves clinically meaningful outcomes. Trials should prospectively assess heterogeneity of treatment effect using predefined patient characteristics and appropriate statistical methods, while avoiding overinterpretation of exploratory subgroup findings.

Where feasible, randomized studies should compare precision-guided strategies directly with contemporary guideline-directed care and evaluate outcomes such as cardiovascular death, recurrent myocardial infarction, stroke, heart-failure hospitalization, and clinically important bleeding. External validation, representative recruitment, adequate follow-up, and assessment of implementation feasibility will be necessary to determine whether these approaches offer meaningful improvements in routine clinical practice.

Ultimately, the next phase of precision medicine in ACS should be defined not by the quantity of patient-level information collected, but by the ability to translate reliable mechanistic, anatomical, clinical, and biological information into safer and more effective individualized care.

## 9. Limitations of the Current Evidence and of This Narrative Review

Interpretation of the proposed framework is limited by heterogeneity in the underlying evidence and by the narrative methodology used to synthesize it.

### 9.1. Heterogeneity and Generalizability of the Evidence

Studies differ in populations, treatment timing, background therapy, procedures, endpoints, and follow-up; findings from STEMI, NSTE-ACS, recent MI, and stable ASCVD are therefore not automatically interchangeable [1,2].

Generalizability is also limited for underrepresented older, frail, multimorbid, and advanced kidney-disease populations. Several abbreviated-DAPT and de-escalation trials were conducted predominantly in East Asian populations [34,37,40,41], requiring attention to population and regimen when extrapolating results.

### 9.2. Phenotypic Precision Exceeds Therapeutic Precision

Imaging, CMR, coronary function testing, and biomarkers increasingly refine ACS phenotypes [7,8,9,16,20,21,22], but phenotype identification alone does not establish a need for different treatment.

This gap is especially relevant to morphology-guided intervention, imaging-defined high-risk plaque, biomarker-selected anti-inflammatory therapy, molecular profiling, and AI-assisted decision support [9,58,59,60,61,71], for which prospective treatment-effect evidence remains incomplete.

### 9.3. Risk Models, Surrogate Endpoints, and Evidence Hierarchy

GRACE, ARC-HBR, PRECISE-DAPT, and the DAPT score were developed for different populations and purposes [30,31,32,72]. They inform risk but are neither interchangeable nor independently prescriptive, and their relevance may change as clinical status evolves [1,2].

Some precision studies also rely on surrogate endpoints. Changes in LDL-C, Lp(a), high-sensitivity C-reactive protein, plaque volume, fibrous-cap thickness, or imaging findings demonstrate biological activity but not necessarily fewer cardiovascular events [52,53,55,56,57,58,60,61].

Indirect comparisons are further limited by differences in eligibility, background therapy, timing, endpoints, and follow-up and should not be interpreted as comparative efficacy.

### 9.4. Rapidly Evolving Evidence and Implementation Challenges

Evidence continues to evolve across imaging, antithrombotic, lipid, inflammatory, cardiometabolic, mechanical-support, and computational strategies [1,2,17,18,19,28,29,33,34,35,36,37,38,39,40,41,49,50,51,52,53,54,55,56,57,58,59,60,61,62,63,64,71]. Preliminary or abstract-only results should be distinguished from peer-reviewed evidence.

Implementation may also be constrained by expertise, infrastructure, cost, and access; applicability therefore requires attention to reproducibility, cost-effectiveness, and equity across healthcare systems.

### 9.5. Limitations of the Narrative Review and Proposed Framework

This narrative review was not designed for exhaustive evidence capture, formal study-level risk-of-bias assessment, or quantitative synthesis. Selection and interpretive bias cannot be excluded, and the review should not be interpreted as a systematic or pooled estimate of treatment effects.

The four-domain framework is an organizing synthesis, not a prospectively validated algorithm. Its components range from randomized, guideline-supported strategies to investigational approaches based mainly on diagnostic, observational, or surrogate evidence; prospective evaluation of the integrated framework is therefore required.

Table 2 provides an integrated summary of landmark clinical studies discussed throughout this review, highlighting the evidence supporting individualized management strategies across the acute coronary syndrome continuum.

## 10. Conclusions

Acute coronary syndromes encompass heterogeneous mechanisms, coronary anatomy, clinical risks, and biological pathways that require individualized management. The proposed four-domain framework integrates these characteristics with revascularization, antithrombotic therapy, and secondary prevention, emphasizing longitudinal reassessment as patient characteristics and treatment priorities evolve.

The principal contribution of this review is its distinction between diagnostic and prognostic precision and clinically actionable therapeutic precision. Although advances in mechanistic phenotyping, coronary imaging, and risk stratification improve patient characterization, their clinical value depends on whether they support treatment decisions that improve outcomes. Accordingly, precision ACS care should distinguish strategies supported by randomized clinical-outcome evidence from those based primarily on mechanistic, diagnostic, prognostic, or surrogate-endpoint findings. The proposed framework remains an organizing synthesis rather than a validated treatment algorithm, and its application should remain grounded in guideline-directed care.

Future research should prioritize prospective studies evaluating whether predefined patient characteristics identify differential treatment benefit or harm. Ultimately, precision ACS care should focus on selecting evidence-based interventions appropriate to individual patients and reassessing those decisions as clinical status, cardiovascular risk, and treatment response evolve.

## Figures and Tables

**Figure 1:**
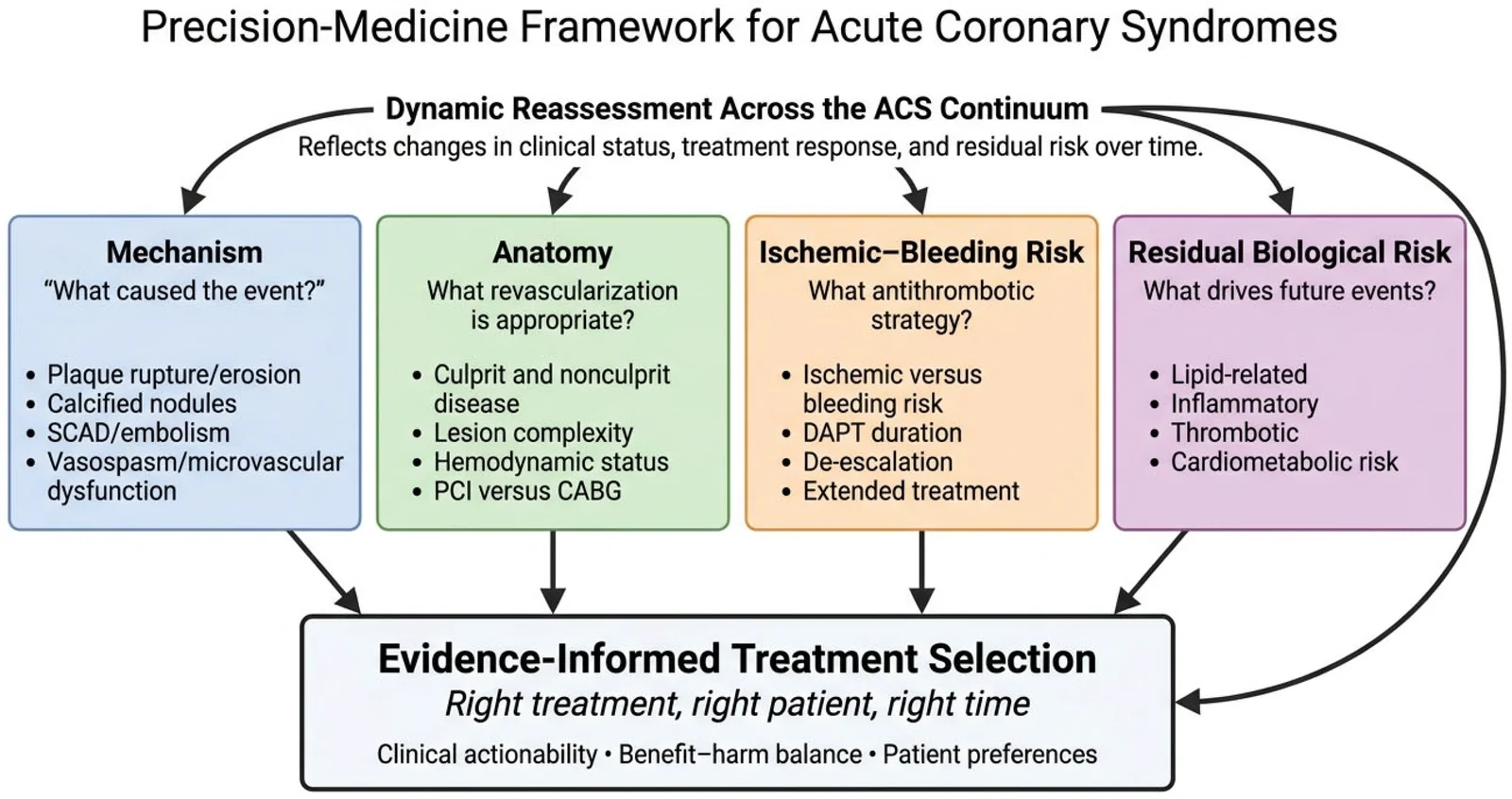
Longitudinal precision-medicine framework for acute coronary syndromes. Four interconnected domains—mechanism of coronary instability, coronary anatomy and revascularization requirements, ischemic–bleeding risk, and residual biological risk—inform evidence-based treatment selection across the acute coronary syndrome (ACS) continuum. Dynamic reassessment accounts for changes in clinical status, treatment response, and residual risk over time. The strength of evidence supporting phenotype-guided interventions varies across domains, and diagnostic or prognostic information alone does not establish treatment benefit. The framework represents an organizing synthesis rather than a prospectively validated clinical decision algorithm. CABG, coronary artery bypass grafting; DAPT, dual antiplatelet therapy; PCI, percutaneous coronary intervention; SCAD, spontaneous coronary artery dissection.

**Figure 2:**
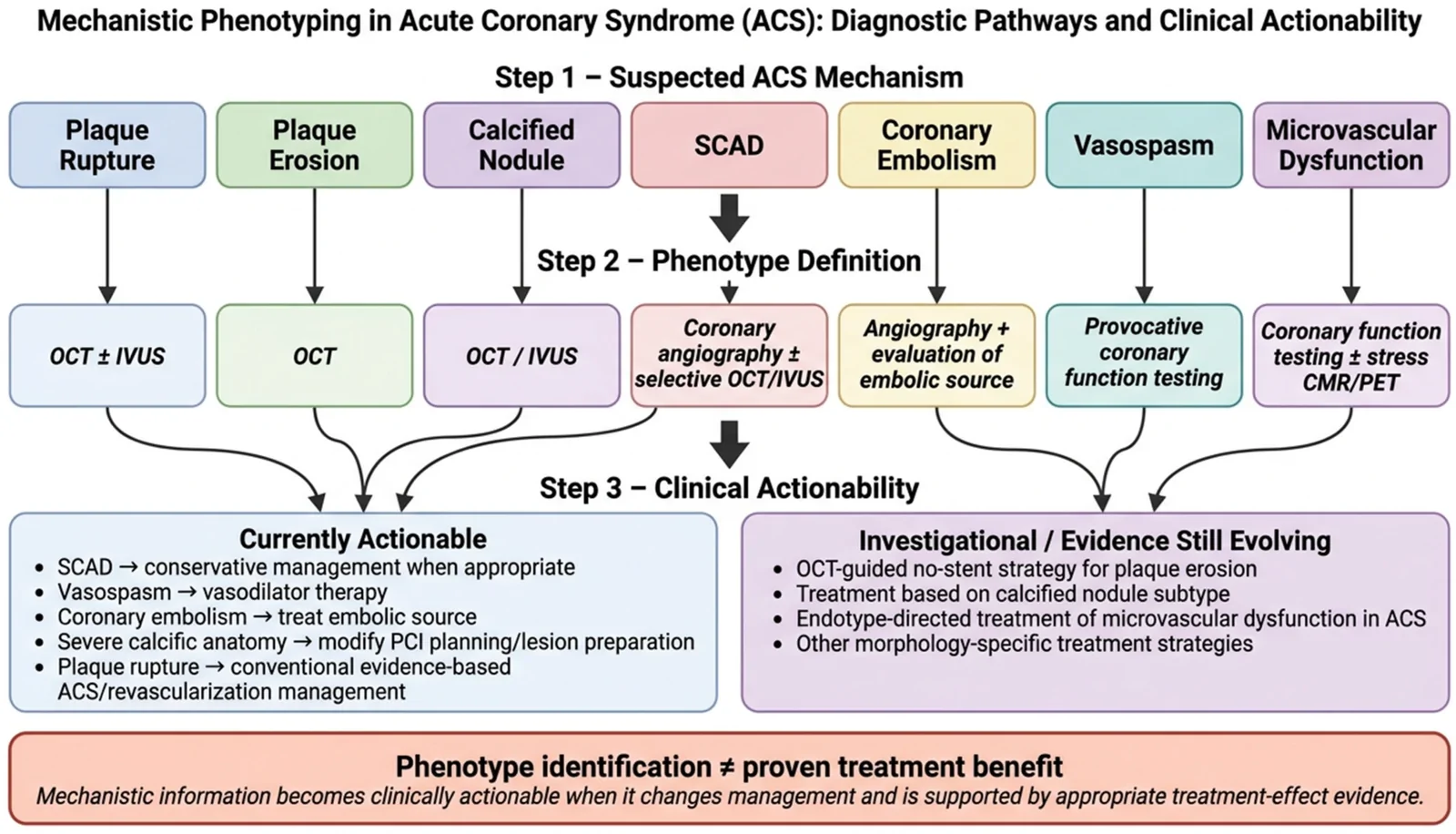
Mechanism-guided phenotyping and diagnostic pathway in acute coronary syndromes. Major mechanisms of ACS are linked to the diagnostic modalities most useful for their identification and to the current clinical actionability of the resulting phenotype. Established management implications should be distinguished from morphology- or endotype-guided strategies that remain investigational or supported mainly by mechanistic, imaging, or surrogate evidence. ACS, acute coronary syndrome; CMR, cardiovascular magnetic resonance; IVUS, intravascular ultrasound; OCT, optical coherence tomography; PCI, percutaneous coronary intervention; SCAD, spontaneous coronary artery dissection; PET, Positron Emission Tomography.

**Table 1:** Practical precision-medicine framework for acute coronary syndromes.

Domain	Key Phenotype	Management Implication	Evidence Considerations
Mechanism	Plaque rupture or erosion; SCAD; vasospasm; embolism; other mechanisms	Identify mechanisms that may alter management and avoid unnecessary interventions.	Management implications vary by mechanism; morphology-guided treatment remains investigational in some settings.
Anatomy	Multivessel or complex coronary disease; cardiogenic shock	Individualize the extent, timing, and modality of revascularization.	Randomized outcome evidence supports selected anatomy- and stability-informed strategies.
Ischemic–bleeding risk	High bleeding risk versus persistent ischemic risk	Individualize antithrombotic regimen, intensity, and duration.	Trial-supported strategies depend on patient eligibility, treatment timing, and drug selection.
Residual biological risk	Lipid-related, inflammatory, thrombotic, and cardiometabolic risk	Optimize established secondary prevention and consider additional therapies according to clinical indications.	Biomarker reduction or prognostic association alone does not establish treatment benefit.
Reassessment	Changes in clinical status, treatment response, and residual risk	Re-evaluate treatment as the patient’s condition evolves.	The integrated framework is an organizing synthesis, not a prospectively validated treatment algorithm.

**Table 2:** Landmark studies illustrating key precision-medicine principles and the strength of supporting evidence across the acute coronary syndrome continuum.

Precision Domain	Landmark Study	Core Message	Evidence Classification
Anatomy/timing	TIMACS [23]	Early invasive management may be particularly beneficial in higher-risk NSTE-ACS; the overall trial did not demonstrate a significant reduction in its primary composite endpoint.	Randomized clinical-outcome evidence; subgroup-based treatment selection
Complete revascularization	COMPLETE [25]	Complete revascularization reduces recurrent ischemic events in hemodynamically stable patients with multivessel STEMI.	Guideline-supported; randomized clinical-outcome evidence
Cardiogenic shock	CULPRIT-SHOCK [27]	An initial culprit-lesion-only PCI strategy improves early outcomes compared with immediate multivessel PCI in selected patients with acute MI and cardiogenic shock.	Guideline-supported; randomized clinical-outcome evidence
Imaging-guided PCI	RENOVATE-COMPLEX-PCI [17]	Intravascular imaging-guided PCI reduces adverse clinical events compared with angiography guidance in selected complex coronary lesions.	Randomized clinical-outcome evidence; guideline-supported in specified settings
Imaging-guided PCI	ILUMIEN IV [19]	OCT guidance improves procedural measures, but the trial did not demonstrate a significant reduction in its primary clinical endpoint.	Randomized trial with a neutral primary clinical endpoint; supportive surrogate-endpoint evidence
Shortened antithrombotic therapy	TWILIGHT [33]	Aspirin withdrawal after an initial period of DAPT reduces bleeding without a significant increase in ischemic events among selected high-risk PCI patients.	Randomized clinical-outcome evidence; guideline-supported in selected patients
High bleeding risk	MASTER DAPT [36]	Abbreviated DAPT reduces bleeding while meeting prespecified noninferiority criteria for relevant clinical outcomes in high-bleeding-risk patients after PCI.	Randomized clinical-outcome evidence; guideline-supported in selected patients
Antiplatelet personalization	POPular Genetics [39]	Genotype-guided P2Y12 inhibitor selection reduces bleeding while meeting noninferiority criteria for thrombotic outcomes in the studied STEMI PCI population.	Randomized clinical-outcome evidence; genotype-guided strategy
Lipid risk	ODYSSEY OUTCOMES [51]	Alirocumab reduces recurrent cardiovascular events after recent ACS in appropriately selected patients receiving intensive statin therapy.	Guideline-supported lipid-lowering approach; randomized clinical-outcome evidence
Mechanistic lipid evidence	PACMAN-AMI [53]	Early intensive lipid lowering with alirocumab favorably modifies coronary plaque burden and composition following acute MI.	Randomized surrogate-endpoint evidence
Inflammatory risk	COLCOT [59]	Low-dose colchicine reduces composite ischemic cardiovascular events following recent MI in the studied population.	Randomized clinical-outcome evidence; guideline recommendations vary
Cardiometabolic risk	EMPACT-MI [62]	Empagliflozin did not significantly reduce the primary composite of first heart-failure hospitalization or all-cause death after acute MI in the overall trial population.	Randomized clinical-outcome evidence; neutral primary endpoint

## Data Availability

No new data were created or analyzed in this study. Data sharing is not applicable to this article.

## Abbreviations

The following abbreviations are used in this manuscript:

ACC	American College of Cardiology
ACS	acute coronary syndrome
AHA	American Heart Association
AI	artificial intelligence
ARC-HBR	Academic Research Consortium for High Bleeding Risk
ASCVD	atherosclerotic cardiovascular disease
BARC	Bleeding Academic Research Consortium
BMI	body mass index
CABG	coronary artery bypass grafting
CI	confidence interval
CKD	chronic kidney disease
CMD	coronary microvascular dysfunction
CMR	cardiovascular magnetic resonance
DAPT	dual antiplatelet therapy
ESC	European Society of Cardiology
GLP-1 RA	glucagon-like peptide-1 receptor agonist
GRACE	Global Registry of Acute Coronary Events
HBR	high bleeding risk
HR	hazard ratio
hs-CRP	high-sensitivity C-reactive protein
hs-cTn	high-sensitivity cardiac troponin
IVUS	intravascular ultrasound
LDL-C	low-density lipoprotein cholesterol
Lp(a)	lipoprotein(a)
MI	myocardial infarction
MINOCA	myocardial infarction with non-obstructive coronary arteries
NSTE-ACS	non-ST-segment elevation acute coronary syndrome
NSTEMI	non-ST-segment elevation myocardial infarction
OCT	optical coherence tomography
PCI	percutaneous coronary intervention
PCSK9	proprotein convertase subtilisin/kexin type 9
RNA	ribonucleic acid
SCAD	spontaneous coronary artery dissection
SGLT2	sodium–glucose cotransporter-2
STEMI	ST-segment elevation myocardial infarction
TIMI	Thrombolysis in Myocardial Infarction
